# Implementation of a sensory modulation intervention in mental health outpatient services: a process evaluation study

**DOI:** 10.1186/s12888-025-07034-5

**Published:** 2025-06-05

**Authors:** Klara Forsberg, Daniel Sutton, Sigrid Stjernswärd, Ulrika Bejerholm, Elisabeth Argentzell

**Affiliations:** 1 Department of Health Sciences, Lund University, Mental Health, Activity and Participation (MAP), Lund, Sweden; 2 Department of Psychiatry, Skåne University Hospital, Region Skåne, Lund, Sweden; 3 School of Clinical Sciences, Auckland University of Technology, Auckland, New Zealand

**Keywords:** Process evaluation, MRC framework, Sensory intervention, Sensory modulation, Occupational therapy, Mental illness, Recovery

## Abstract

**Background:**

Mental health service users often experience difficulties interpreting and regulating sensory stimuli resulting in increased anxiety, decreased abilities to engage in activities and a hampered recovery process. However, there are limited studies on the implementation of such recovery-oriented interventions targeting sensory difficulties via sensory modulation techniques. Therefore, the aim of this study was to investigate staff and manager views on the implementation process of a group-based sensory modulation intervention in mental health outpatient services in Southern Sweden.

**Methods:**

This mixed method implementation process evaluation included eight outpatient units, which were also study sites for a Randomized Controlled Trial (RCT) (NCT06432114), evaluating the effectiveness of the sensory modulation intervention. Quantitative data were analysed using descriptive statistics and qualitative data were analysed using deductive and inductive content analysis.

**Results:**

The results indicated that the intervention was highly accepted by the mental health staff. The dose delivered and received were high and the intervention in general met the needs of the target group. Managers and staff reflections indicated that following the intervention service users seemed to feel better prepared to handle anxiety in daily life due to the acquisition of new sensory coping strategies. Staff expressed that they benefitted from acquiring a different perspective or “new sensory glasses” to apply in their clinical practice. However, managers’ and staff reflections also highlighted the need for an adapted manual for people with cognitive issues and more education for staff.

**Conclusions:**

This study contributed to new knowledge of implementing a recovery-oriented sensory modulation intervention in mental health outpatient services. The implementation was generally carried out as intended. Nonetheless, certain challenges emerged during the implementation process, both within the contextual environment and during the delivery of the intervention.

**Trial registration:**

Retrospectively registered 20,240,529, in ClinicalTrials.gov NCT06432114.

## Background

Sensory processing is central to human experience, affecting our ability to function and relate to others and directly influencing our mental health [1, 2]. Sensory modulation is the aspect of sensory processing related to regulating and adapting to stimuli. This involves both neurophysiological mechanisms within the central nervous system and related behavioral responses that act to regulate the type and intensity of stimuli being processed [3]. Evolving research shows that persons living with mental illness have more difficulties processing and regulating sensory stimuli than persons without mental health issues [2, 4, 5]. Potential difficulties related to hyper or hyposensitivity to sensory input, include increased anxiety affecting engagement in daily activities [6] as well as the recovery process [7].

Over recent decades mental health services have moved towards a more recovery-oriented approach with the aim of better meeting the needs of service users and supporting them to be the agents and experts of their own mental wellbeing [8–10]. Interventions targeting self-management and engagement in meaningful daily activities are considered recovery-oriented interventions [11]. Sensory modulation interventions assist with self-management of physiological arousal, emotional and behavioural responses, via specific calming or alerting sensations (including visual, auditory, tactile, proprioceptive, vestibular and olfactory input) [12]. Furthermore, group-based interventions that facilitate connection and include informal peer support have also shown to support recovery [13, 14]. The evidence base for recovery-oriented interventions targeting sensory processing issues for mental health service users has mainly been developed in inpatient services targeting more acute issues and the reduction of coercive practices. Reviews on such interventions have shown effects of decreased restraints and helping service users self-regulate and calm themselves in acute episodes [15, 16]. Research on sensory modulation intervention within outpatient units is scarce. However, a few existing studies indicate the potential for positive outcomes such as reduced anxiety [17], experiences of improved participation [18] and increased engagement in daily life activities [19].

Process evaluation provides a useful framework for exploring the implementation of new complex interventions, such as a group-based sensory intervention, within mental health services [20]. Complex interventions can be defined as interventions that involve multiple aspects or components, including different settings, different health issues and high flexibility of the intervention [21]. When evaluating the effect of such interventions using RCT design, process evaluations can help explain the reasons for different outcomes and make the clinical application more relevant [20]. Recently conducted process evaluation studies have focused on complex group interventions targeting daily life for mental health service users, that were implemented in mental health outpatient units. Several factors in the implementation processes of these interventions were reported to potentially affect the outcomes of the larger trials investigating intervention effectiveness [22–24]. For example, one study investigating service user perspectives, reported that feeling safe and supported in the group affected the number of service users who completed the intervention [22]. A study investigating service provider perspectives pointed out that the team members’ positive attitudes towards the intervention facilitated the recruitment of service users and was an important implementation factor [23]. A further study reported that staff being provided with detailed delivery information in a manual was shown to be important for successful implementation [24]. An implementation study conducted within inpatient mental health units found that the introduction of sensory approaches were associated with positive changes in ward climate, better relationships between staff and service users, and decreased seclusion hours per year [25].

However, to our knowledge, there are no studies examining implementation of a group-based sensory modulation intervention for mental health service users. Therefore, this present process evaluation study was designed to explore relevant implementation components in context and understand mechanisms of impact to better explain the outcomes within a larger trial (Reg.nr: NCT06432114), which evaluated the effectiveness of a group-based sensory modulation intervention in mental health outpatient units in southern Sweden [20].

Specifically, this study aimed to investigate staff and managers’ perspectives on the implementation of a group-based sensory modulation intervention in Swedish mental health outpatient units. Service user perspectives of the intervention and its impact have been published elsewhere [19].

## Methods

This mixed-method process evaluation study evaluated the implementation of a sensory modulation intervention in mental health outpatient units in southern Sweden. The Medical Research Council (MRC) framework for process evaluations of complex interventions was used to guide the process [20].

### Research setting

This study was conducted over 14 months following a multi-center Randomized Controlled Trial (RCT) (Reg.nr NCT06432114), which evaluated the effect of a sensory modulation intervention in sixteen mental health outpatient units in three different regional psychiatry organisations in southern Sweden. The overall aim of the RCT was to investigate the effectiveness of a sensory modulation intervention compared to treatment as usual (TAU) among 200 service users in mental health outpatient units. The RCT hypothesised that the Sensory Awareness Program (SAP) would be more effective than TAU in terms of reduced anxiety (primary outcome) at three months follow-up. Secondary clinical and personal recovery outcomes were also measured at post-intervention, and three and six months follow up, and assumed to be in favour of the intervention group.

In this current process evaluation, eight mental health outpatient units from one psychiatric organisation took part, including four general psychiatry units and four psychosis units. Half of the units were in a more urban area. All service users who participated in the intervention, within the outpatient units, had a psychiatric diagnosis such as Psychosis, Depression, Anxiety syndrome or Post Traumatic Stress Disorder. The mean time of contact with psychiatric services was nineteen years. Please see related details in the reach section of the results. The staff included in this process evaluation study were eight first-line managers and sixteen staff who delivered the intervention.

### Intervention

#### Sensory awareness program (SAP)

The following description of the intervention aligns with the Template for Intervention Description and Replication (TIDieR) checklist [26]. The Sensory Awareness Program (SAP) is based on sensory modulation theory and practice, serving as an adapted version of the American manualized group intervention, the ‘Sensory Connection Program’ [27]. SAP is an 11-week, manual-guided intervention aimed at equipping participants in mental health services with individualized sensory strategies to help regulate physiological arousal linked to anxiety. The intervention was conducted face-to-face in a group format, accommodating up to 8 participants per group. The program began with a one-on-one session for each participant to complete the sensory profile questionnaire [28]. This initial session introduced the concept of sensory modulation and allowed participants to discuss personal sensory needs and preferences. Following the individual session, participants attended weekly group sessions for 10 weeks, with each session lasting between 1.5 and 2 h.

The purpose of the intervention is to support participants in identifying daily triggers, understanding their unique sensory profiles, and applying sensory strategies in challenging situations to reduce anxiety and enhance engagement in daily life.

The group sessions included psychoeducational content and exercises focused on exploring and practising sensory strategies, to apply both during sessions and at home. Examples of sensory strategies included the use of headphones to mitigate auditory-related distress, a weighted blanket for anxiety relief through deep pressure, and physical exercises for proprioceptive stimulation to enhance alertness. An essential component of the intervention was the setting of individualized goals related to managing overwhelming anxiety and the development of coping strategies for handling daily activities.

Each group participant received a manual containing worksheets and exercises, while course leaders were equipped with a detailed instructional manual to ensure a structured and consistent delivery of the program. The course leader who led the intervention was an occupational therapist, who collaborated with co-therapists such as nurses, psychologists, and social workers from the outpatient team. Before delivering the program, course leaders and co-therapists underwent a two-day training in sensory modulation and the program content.

### Implementation program

A structured program was used to guide the implementation of the intervention in the units. The process evaluation was completed over 14 months, with the intervention being delivered in different units sequentially. The first unit started implementation in April 2022 and the last unit ended in June 2023, alongside with the RCT project. Two trained staff (one occupational therapist and one co-therapist) delivered the intervention in each unit, and all staff remained the same during the entire implementation period. See Table 1 for details.

**Table 1:** Description of the implementation program

Components of the implementation program	Content
Piloting first version of SAP 2018–2020	- The testing of pilot version of SAP and adaptions made of the manual before the RCT project started.
Introductory phase RCT project 2021–2025 Process evaluation (14 months) April 2022– June 2023	- Introduction to the concept of sensory modulation for managers and staff in outpatient units
Preparatory phase	- Decision by managers in mental health services and user organization to implement/test SAP in outpatient care - Providing education platform for staff to use in their education
Education phase	- Education in SAP concepts for course leaders and co-therapists at nine outpatient units (eight remained in the project after education)
Data collection - RCT step 1	- Data collection, before allocation and delivery phase, digitally and on paper
Delivery phase	- SAP-groups held for 11 weeks in each unit
Data collection - Process evaluation	- Interviews beginning of delivery phase (managers and staff)
Continuous supportive feedback	- Weekly supervision with PI of SAP, for course leaders and co-therapists
After intervention delivery phase - Data collection - RCT step 2–4 - Data collection - Process evaluation	- Data collection digital and on paper directly after intervention, 3 months after and 6 months after delivery phase (Not possible to keep doing/spreading SAP according to RCT-design) - Interviews directly and 6 months after delivery phase (managers and staff)
SAP- delivery phase for control group according to waitlist-design	- Each unit doing SAP with control group after the last data collection is done

### Process evaluation plan

This process evaluation plan was designed by the fourth author (UB) based on the MRC framework [20], which included the components of context, implementation, and mechanisms of impact. Please see Table 2 for details of the plan.

**Table 2:** Process evaluation plan of SAP

Components	Description	Questions	Data sources*
Context	Contextual components that may influence implementation	- What barriers and facilitating components exist on local and operational levels of the organisation and interact and impact on implementation of the intervention? - To what extent was SAP implemented as planned?	- Individual interviews with first-line managers - Focus group interviews with staff delivering the intervention - Fieldnote documentation from supervision
Implementation	Delivery of the intervention, i.e. what and to what extent, how much and to whom	- To what extent was SAP implemented as planned? - How much, when and what was delivered by SAP staff as proposed? (i.e., introduction, delivery phases and sessions) - How much, when and what in the delivery, i.e., introduction, delivery phases and sessions benefitted participants? - To what was the target group of SAP reached as proposed? - What adaptions were made to enhance the fit of the intervention to the context?	- Fidelity scale items directly after delivery - Individual interviews with first-line managers - Focus group interviews with SAP staff delivering the intervention - Fieldnote documentation from supervision - Incident documentation (adverse events)
Mechanisms of Impact	Responses and reflections of the intervention, and mediators that influence change	- What are the responses to the intervention and mediators that impact change of behaviours and outcomes?	- Fidelity scale items directly after delivery - Individual interviews with first-line and middle managers - Focus group interviews with SAP staff - Fieldnote documentation from supervision - Incident documentation (adverse events)

*If not stated otherwise data sources was collected according to RCT– structure. Around baseline (1–2 weeks) of delivery, directly after Sap was delivered and at

6-months follow-up, except fieldnotes and incidents which was documented weekly during supervision meetings of 10 weeks

### Recruitment

Participants were recruited through purposeful sampling and were staff (course leaders and co-therapists) who provided the intervention and managers at the mental health outpatient units. During the first two years of the RCT study, all possible staff and managers were asked by the last author (EA) to participate in interviews and all consented and filled in written consent forms.

Most managers and staff had significant experience of working in mental health services, and were experienced in delivering group interventions, though most of them were not experienced in delivering sensory modulation interventions. The managers consisted of three men and five women. The staff were all women and included eight course leaders (occupational therapists) and eight co-therapists (two occupational therapists, two social workers, two nurses, one care assistant and one psychologist).

### Data collection

Data collection included both quantitative data from the RCT data collection and qualitative data collected through individual interviews with managers and focus group interviews with staff.

Qualitative data collection was completed with managers, course leaders and co-therapists before, directly after and six months after the intervention ended. The third author (SS) conducted the interviews with managers, and the last author (EA) facilitated the focus-group interviews.

Managers were individually interviewed through telephone interviews. Course leaders and co-therapists participated in focus groups before the program (via online platform), immediately after the program (in-person at a university setting) and six months after delivery (via online platform). The interview guide was semi-structured and developed by the fourth author (UB) based on the components in the MRC framework [20] and was used both for the individual and focus group interviews. Examples of questions were “*What is your general impression of sensory modulation and the intervention? ”*,*“Do you think the intervention fits into your treatment program here at the unit?”*,* “Do you feel ready to be a course leader? ” and “Please describe the target group at your unit and those who participated in the intervention?”*,* “Was there anything in the intervention that could not be implemented?”*,* “Do you feel that anything has changed in your unit overall during the time you have been providing the intervention? If yes*,* in what way?”*

Qualitative data also included documents in terms of field notes collected during the delivery phase by the supervisor (last author).

Quantitative data included dose delivered/received and reach, which were collected within the RCT data collection, mainly digitally through the data management system Redcap [29]. A small portion of the data were collected manually with pen and paper for those service users who preferred this. Data collection of fidelity was filled in by the staff directly after their delivery of the intervention. The fidelity scale used in this current study was developed based on a fidelity measure of Ayres Sensory Integration intervention [30] and consisted of general questions and a detailed section with ratings on a Likert scale from one to seven. In the current study, one general question was included; *I think we have implemented the sensory modulation intervention exactly as we planned to do?* And six content questions covering details such as if service users received knowledge of sensory processing and if they had been supported to explore their sensory profiles and set individual goals.

### Data analysis

All qualitative interviews were transcribed verbatim and analysed deductively through the Medical Research Council (MRC) framework [20] for process evaluations using deductive content analysis according to Elo and Kyngäs [31]. The first author started the analysis process by reading and listening to the interviews to receive a sense of the whole of the data. Meaning units related to the aim were coded and categorised by the first author (KF) according to the analysis matrix based on the MRC framework [20] with the categories of context, implementation and mechanism of impact. The last author (EA) took part in this analysis process in an iterative process and all the meaning units, codes and categories were validated and discussed with the last author for consensus (EA). In the next step, the fourth author (UB) did further analysis focusing more deeply on the components of implementation followed by the third author (SS) and second author (DS) who also did further analysis of the material. This collaborative process between the authors led to changed categories and resulted in a conceptual map of important factors in the implementation process of the intervention. Following the deductive analysis mechanisms of impact including mediators were further analysed with manifest inductive content analysis [32], to gain a deeper understanding of the real impact of the intervention. The inductive approach started with the first author (KF) reading the meaning units in the deductive category of mechanisms of impact. The meaning units were further condensed, labelled with new codes, further analysed and categorized into sub-categories related to the impact of the intervention on staff and service users. The inductive analysis process was completed in a collaboration between the first (KF), second (DS) and last author (EA). The quantitative data were analysed using descriptive statistics to measure the dose delivered/received, reach and fidelity during implementation.

## Results

The result showed that the sensory modulation intervention in general had a high acceptance among the managers and staff who were positive towards the intervention and believed the implementation program could be completed as intended. However, implementation challenges also emerged. Please see Table 3 for an overview of important components of the implementation process.

**Table 3:** Important components of the implementation process

Context	Implementation	Mechanisms of impact Sub-categories and critical mediators
Positive attitudes and supportive teams	High attendance of service users	Increased awareness improved preparedness to handle anxiety
Engaged and experienced course leaders	SAP fits well early in the treatment process	Service users’ cognitive level potentially affected their ability to take home strategies
Professions-specific intervention was encouraging	More extensive education was asked for to be better prepared for delivery	New “sensory glasses” in clinical practice
Good fit and a hands-on intervention complementing talking therapies	Supervision with PI did ease the delivery	Positive implementation climate
RCT-design of the broader study created difficulties in recruitment process	Reached out to a diverse age group of service users	Structured intervention and supervision
	New theory behind the intervention which took time to learn for staff	Recovery-oriented approach

### Context

Various contextual barriers and facilitating factors affected the implementation of the intervention. In the early phases of the implementation process, there seemed to be a positive implementation climate in the different outpatient units. This was shown by several different facilitating factors and a generally positive attitude toward research in general and the implementation of the intervention specifically. Staff delivering the intervention reported that their colleagues and managers at the units were in general highly supportive of the course leaders and co-therapists delivering the intervention.

*He [the manager] is very positive about groups*,* and he cares so much about patients with psychosis. He hasn’t questioned anything*,* and he’s very positive about it [the intervention] now when he knows we’re doing this. /Staff 3*.

This positive climate remained during all parts of the implementation. The managers and staff in several units were not only positive about the sensory approaches, but they also had experience with implementing new group interventions and were positive towards this format. Groups was viewed as beneficial both in terms of therapeutic outcomes for service users, but also in terms of efficiency.

*Yes*,* and the fact that it’s a group treatment*,* we need to have group treatments since we need to be able to handle more patients at a time*,* to be efficient*,* so that’s positive. /Manager 3*.

Another contextual facilitating factor was that several managers had pre-knowledge and earlier positive experiences of the researchers, and the research network to which the intervention adhered. Technical and administrative support in terms of rooms and technical aids for delivering the intervention was provided in all units, demonstrating that the physical environment was also an implementation facilitator.

However, for most managers and staff, sensory modulation was a new theory base and approach. This created great interest in testing the intervention and the course leaders also appreciated that it was a profession-specific and manualized intervention, providing a theory base and structure for delivery.

*Because it is occupational therapy and a slightly different occupational therapy approach*,* it would be great if it could be part of our program at the clinic. /Staff 9*.

Staff and managers further reflected on the benefits of the intervention as a good fit for the target group in their units. This was due to their belief that service users often do not understand bodily reactions to stimuli from the environment, which was viewed as a barrier to the possibility of engaging in daily activities.

Additionally, several managers believed that the new intervention complemented other standard treatments within both general psychiatry and psychosis units. The sensory intervention was perceived as more “hands-on”, complementing talking therapies for anxiety symptoms such as Cognitive Behavioural Therapy (CBT).

*Predominantly we have communication through language and with a CBT focus*,* which I advocate as well*,* but some people have difficulty defining and formulating their anxiety*,* which is so strong… it’s so clear that it’s physical… and then to become aware of what is happening to me in more aspects. /Manager 5*.

Six months after the program delivery all managers thought that having engaged staff was the most crucial contextual factor facilitating implementation. In several units, there were also contextual changes in the awareness and potential use of sensory interventions six months post implementation. This was shown by the fact that the occupational therapist received referrals from colleagues regarding the provision of sensory aids and additional sensory interventions for service users.

There were also contextual barriers during the implementation. One of these was the program recruitment process, which was experienced as both demanding and time-consuming for the staff, who already had a high workload. There was also a discrepancy between the units, where most general psychiatry units had an easier process of recruitment than most of the psychosis units. The teams in most units were supportive and seen as a facilitator, but there were some units where this was the opposite. The latter was viewed as a contextual barrier negatively affecting the recruitment and program attendance of service users.

The RCT design of the wider project was also viewed as a barrier to the recruitment process since the service users were less motivated to take part in a research project where they were not guaranteed to receive the intervention. Even though the project had a waitlist design, the risk of a long wait until data collection ended was a barrier to recruitment.

*Because it was a study*,* and you were randomized and didn’t know which group*,* some people thought it was exciting*,* while others may have thought that it wasn’t worthwhile… that they declined because they didn’t know whether they would be included. /Staff 12*.

However, six months after the intervention ended staff reflected that recruiting group members in the future would be easier to manage since they felt more confident in explaining the content of the intervention to service users and colleagues. A further contextual barrier reported was staff being on sick leave, which prolonged the delivery phase in a couple of units.

### Implementation

In general, the intervention was implemented as intended, which was reflected in the fidelity scale evaluation [30]. According to staff ratings there was a good implementation fit, with a mean value of 5.6 in the overall fidelity question (min. 5 - max. 7). In several units the service users and staff adapted to the program easily. However, in a few units, the implementation was more challenging as elaborated on below.

### Dose delivered and received

Most service users were eager to attend all sessions, which resulted in very low dropout rates across all groups. The extent to which the intervention could be delivered as intended, the dose delivered, was 100% in all units. The dose received among service users varied between the units with the lowest in a psychosis unit being 60% of the service users who started and completed the entire program and the highest in a general psychiatry unit being 100%, and the total dose received in service users within all units was 80%. In the six fidelity questions related to the detailed content delivery, the mean value range was 4.9–6.5. The timing of the intervention delivery generally aligned with early intervention, as it was offered at the beginning of service users’ treatment. Staff reported that the intervention could be useful when someone was severely ill and could be a facilitator for attending further interventions, such as individual talking-based therapies. Another suggestion was that the new intervention could be delivered as part of trauma programs, where service users would benefit from learning sensory based and body-centred strategies, before attending exposure therapies.

*We have discussed within the team… that it could be a good intervention as preparation for patients who are going to undergo trauma treatment… the initial phase in those therapies usually goes a long way to finding strategies to ground*,* so that you don’t dissociate or that the anxiety becomes too high during the exposures… that you may need several stabilising interventions before. /Staff 8*.

The required preparation before and during the program delivery was experienced as intense. The reflections of the staff also indicated that the two-day education, the manual and the literature were not enough information for them to feel confident in the delivery of a novel intervention with a new theoretical base.

*I think even if it’s a study*,* you still want to give a professional impression to the group*,* and they also… expect something to be delivered that gives quite a lot back….so yes*,* a lot of preparation… you want to feel safe…during the session. /Staff 6*.

However, the supervision that was provided through all parts of the delivery phase was viewed as a facilitator and increased the staffs’ capacity and confidence to deliver the intervention.

The RCT data collection during the implementation was reported by the staff to be acceptable for most service users. Although the staff reported that some service users found it to be cognitively tiring, especially within the psychosis units. The data collection within the research project was also mentioned as increasing the administrative workload for staff.

In the first sessions of the delivery phase most staff expressed that the manual contained a lot of information in relation to the number of sessions. However, the content was experienced as important knowledge for service users’ mental health, and staff made great efforts to understand the new theory so they could deliver the content effectively. At the end of the program delivery staff had managed to facilitate the group intervention and believed that delivering the next group would be easier. Several units wanted to continue providing the new intervention in the unit with the ambition to more thoroughly implement it in the unit’s standardized programs.

*I think it’s a good material and… we will be able and want to work with it [the intervention] again… maybe change it a bit…adapt the theory a bit and focus on practical [strategies]. /Staff 14*.

To address delivery issues staff made minor adaptions to enhance a better fit. These minor changes were done in dialogue with researchers and experts on sensory modulation during weekly supervision.

Staff had suggestions for improvement of the manual to meet the needs of different service user groups. Staff providing the intervention in the psychosis units generally suggested larger adaptions, while the service users in general psychiatry could receive most of the content in the version delivered. One suggestion for improvement was that the manual could be structured with basic content and then additional advanced content for facilitators to offer as needed or wanted by group members. Further suggestions were to add more sessions and to facilitate the sessions with more power-point lectures, as well as to remove some of the worksheets to minimize the workload for service users during some sessions.

### Reach

In general, the intervention reached the intended target group, including 50 service users. Half of the units were for people with psychosis and half were general psychiatry units. Services users had a diversity of diagnoses such as Psychosis, Bipolar disorder, Neuropsychiatric disabilities, Depression, Anxiety syndrome and Post Traumatic Stress Disorder (PTSD). There was a mix of both younger and older service users, the range was between 23 and 77 years old with the mean age being 46 years old. Participants contact with mental health services was varied, with the range from 1 to 52 years and a mean time of 19 years. Women (74%) were overrepresented as well as service users born in Sweden (96%).

Reach appeared to be easier to achieve in some units than in others, for example in contexts where the intervention was delivered to service users assessed and already in line for CBT.

*I got a lot of help from those who have a little overview of our waiting lists*,* a psychologist came and said she looked through the waiting lists to pick patients and my co-therapist who is a social worker said I can go and look… I thought it would be much harder to get participants*,* but it felt like the clinic came together… So*,* I didn’t have to do much to get the participants. /Staff 1*.

In psychosis units in general, the outreach was more difficult than in general psychiatry. Staff described having problems with motivating service users to attend a new unknown intervention and in some units, it was organisational barriers, such as large units or collaboration issues.

### Mechanisms of impact

The staff and managers had several reflections on the impact of the sensory modulation intervention, including reducing service users’ symptoms and changing clinical practice. These mechanisms can be divided into three subcategories; *Increased awareness improved preparedness to handle anxiety*,* Service users’ cognitive level potentially affected their ability to take home strategies and New “sensory glasses” in clinical practice.*

Staff described at the beginning of the delivery of the intervention that after the early sessions, it seemed to have started “something” within the service users, which was explained as a new understanding of different sensory experiences within their bodies. This increased service user awareness was reported by staff as remaining throughout the entire delivery phase. Six months after the intervention was delivered staff reflected that this change in how the service users experienced their bodily reactions was still present. Staff further reflected that service users seemed to have added behaviours and coping strategies connected to sensory modulation which seemed to positively impact daily living in terms of being better prepared to handle anxiety situations and feeling safer. Managers and staff expressed that several service users reflected that they were surprised about the impact the sensory intervention had on their daily lives. The new knowledge of bodily reactions and increased ability to regulate sensory experiences helped to prevent anxiety attacks.

*The client expressed “Now I understand these things that can trigger my anxiety*,* I’ve never understood that before. I’ve just had some discomfort so*,* not been able to think about it.” So*,* a lot of things landed for that particular client. /Staff 1*.

A further impact reported was that service users could use sensory strategies to endure a range of psychiatric symptoms, including auditory hallucinations.

*They were positive about being able to find things they could do for their problems*,* so to say*,* if it was very troublesome voice hallucinations*,* to use headphones and so on*,* that it had helped. They could try out*,* what could help. /Manager 2*.

However, in some units, some of the content in the intervention seemed to be experienced as triggering, especially at the beginning of the delivery where sensory triggers were specifically discussed in the group. A few service users seemed to have an increase of anxiety at this point, which required extra support from staff during and between sessions.

According to both managers and staff the service users’ cognitive function and fatiguability affected their ability to receive the entire intervention. This seemed to be related to the type of mental health issues service users were experiencing, as those with psychosis struggled more than service users with mood disorders and anxiety in the general psychiatric units.

*I have also realised that it costs a lot of energy. I think it has to do with the patients’ functional level*,* so not everyone has such a good functional level here and if you have a slightly better functional level*,* I think you can utilize it (the intervention) better. /Manager 8*.

Managers and staff expressed that difficulties with tiredness seemed to impact the ability for the service users to “take home” and implement coping strategies acquired during the intervention.

Delivering the intervention impacted the course leaders’ practice in that they expressed that they had received so-called “new sensory glasses” and a new sensory-related language.

*The occupational therapists… they have this knowledge with them to a greater degree when they meet patients in everyday life*,* and this is taken into account… I think that you are wearing some new glasses. /Manager 6*.

This was described as affecting how they perceived service users’ challenges and needs, with greater awareness of potential sensory difficulties affecting service users’ daily lives.

### Mediators

Several critical mediators for change were identified, including *Positive implementation climate*,* Structured intervention and supervision* and a *Recovery-oriented approach.* One critical mediator for change was a positive implementation climate, with staff having high levels of acceptance and positive attitudes towards the intervention in most units. Another key factor for change seemed to have been the structure of the sensory intervention and the fact that it was a manual-based intervention. Supervision from the research team was also a potential mediator for change since it seemed to have eased delivery by providing support and guidance to group facilitators.

A unique aspect of the intervention, reflected on by managers and staff, which also differed from other interventions provided at the units, was that it seemed to include an empowerment aspect. The program directly focused on what the service users themselves decided was a good fit for them, including their goals and which coping strategies they used. Furthermore, the recovery-oriented approach with a focus on mental wellbeing and compassion, rather than illness, along with the group-based format with informal peer support, seemed to be a strong mediator for change throughout the program delivery.

*So*,* they shared very nicely and helped each other a lot*,* and really listened*,* I felt*,* with a lot of respect for each other’s concerns. /Staff 12*.

## Discussion

This study contributed new knowledge about staff and managers’ views of implementing group-based sensory modulation interventions in mental health outpatient services.

The findings showed that the implementation at units in general was performed as intended with good general fidelity, high dose delivery (100%) and high dose received (80%). Our result further showed that a positive implementation climate and supportive teams in most units was a strong contextual mediator which facilitated change. This is aligned with earlier research, which concluded that an important factor when implementing sensory modulation interventions successfully was supportive managers and teams [33].

A finding in the current study was that it seemed to be highly motivating to both deliver and receive the new intervention. The intervention was viewed as something the staff in mental health services thought was worth the effort to implement, although the workload was high in learning new theories and properly applying them throughout the delivery. One potential reason for this motivation within staff could be that they believed the intervention improved their ability to help service users towards better mental health. They reported that the intervention supported reductions in psychiatric symptoms, such as anxiety, and increased service users’ ability to engage in activities of daily life. An earlier study within the larger project of this current research, investigated service users’ experience of participating in the intervention and highlighted similar findings. The participating service users reported increased bodily awareness and a stronger sense of self, as well as new tools to handle and plan daily activities according to their own needs [19]. Recent research has shown similar results when implementing sensory modulation interventions in inpatient units. Azuela et al. [25] showed that staff experienced improved confidence and strengthened relationships when supporting service users to self-manage distress and understand sensory issues. Another study by Björkdahl el al [34] reported that implementing sensory rooms in inpatient units improved the staffs’ ability to help service users towards emotional calmness. The sensory tools appeared to increase the service users’ sense of empowerment, which motivated staff to deliver the intervention. Further, in line with the result of the current study, research on staff experience of implementing sensory strategies in inpatient units found that staff expressed a need for more education in sensory modulation to support delivery and increase effectiveness for service users [35, 36].

The intervention implemented in the current study was profession-specific and hence led by occupational therapists, although co therapists were of other professions. The occupational therapists appreciated that it was a profession-specific intervention since it was viewed as strengthening the profession, as also reported in other studies on profession-specific interventions [37]. Additionally, the intervention was viewed as hands-on and strongly related to activities in daily life, which was important for the service users who attended, as shown in earlier research in this project [19]. Further, course leaders experienced that their new knowledge of sensory modulation extended and improved their clinical practice. However, the implementation process, in terms of the possibility to continue delivering the intervention after the research ended, seemed to be negatively affected by the intervention being profession-specific since the number of occupational therapists in Swedish mental health services is limited. Wright et al. [38] reported similar issues in an Australian context when implementing a sensory modulation intervention in mental health inpatient units. They suggested that successful implementation should include the involvement of all professions in mental health services, guided by an occupational therapist.

The fact that clinicians had no earlier training in sensory modulation theory and that the current sensory modulation intervention was a new type of rehabilitation concept in Swedish mental health services added several implementational challenges. This is not surprising, but the current study produced valuable findings, highlighting factors that facilitated implementation and reduced barriers. These factors included positive implementation climate and engaged staff, a structured manual, and supervision during delivery from researchers, which eased the implementation of the new intervention. This adds important knowledge about how to assist optimal delivery in future research projects and clinical practice. To our knowledge, there are no previous studies that have investigated factors affecting the implementation of sensory modulation group interventions in outpatient units. However, implementation research in other new recovery- and group-based interventions in mental health outpatient units highlight several important facilitators for successful implementation. In line with the results in the current study, these facilitators included positive attitudes and collaborative climate in units which affected reach in a lifestyle intervention [24] and having a structured intervention which was crucial for an optimal delivery process [23]. In the current study, the informal peer support that developed within the intervention groups, was also an important mediator for change during the implementation process in terms of empowering service users. This phenomenon has been seen in earlier research in outpatient units studying the implementation of group interventions [39, 40].

Measuring the effectiveness of interventions using RCT design can add evidence and contribute to best clinical practice. However, this current study showed that the strict design came with problems with recruitment affecting the outreach, as well as adding heavy administrative work for staff in their already high workload in mental health services. A recent systematic review of process evaluation studies attached to pragmatic RCTs highlighted several important considerations, such as using process evaluations to better explain and apply results from trials to make improvements in interventions [41]. The process evaluation data in this current study add new knowledge about the implementation of a group based sensory modulation interventions in outpatient units. This knowledge could potentially be of clinical value for psychiatric teams when implementing new interventions in general, but especially when implementing interventions targeting service users’ self-management, which aligns with the goal of increasing recovery-oriented care.

### Strengths and limitations

The strengths of this study include that the data were rich and diverse, with several types and sources of data included, which increases the validity and trustworthiness of the findings [42]. Additionally, the data collection was over one year, providing a longitudinal perspective of the influencing factors and impact through pre-delivery, delivery and post-delivery phases. The participants were both managers and staff delivering the intervention, hence the findings cover data from those closest to program delivery as well as those with knowledge of the organisational context. This allowed for triangulated sources and strengthened the findings. There was also triangulation in the analysis as several researchers collaborated in the process, which further increased reliability and trustworthiness [43]. Both general psychiatry and psychosis units were included, and the unit locations were in varied geographical areas (urban/rural). This increased the range of mental health service users participating in the group program and supports the transferability of findings across mental health contexts [32].

There were also study limitations. The data did not cover the entire RCT population, which might affect the validity of the findings. However, collecting data over the whole period of the larger trial was not administratively possible. Further, there was no process evaluation data included from the service users since the voices of the service users receiving the new intervention have been reported in an earlier study [19]. However, including mental health service users might have increased the trustworthiness in terms of further triangulation between sources and offered different insights, based on experience-based knowledge of receiving of the intervention [9]. Furthermore, the staff, managers and service users within the group interventions were not gender diverse, with the majority being women. This potentially affects the generalisability of the findings across a wider population.

## Conclusions

The process of implementation investigated in this current study showed that the sensory modulation group program was seen as an accepted treatment by managers and staff delivering the intervention in Swedish outpatient mental health services. Even though the implementation required a heavy workload all units included in this process evaluation managed to successfully deliver the intervention with high levels of fidelity. All participants reflected at six months post-intervention, that they planned to deliver the intervention again in their units. However, we suggest adaptions in terms of a prolonged education before the delivery and that the manual could be revised with the staff included in this current study as well as mental health service users, to improve future delivery of this sensory modulation intervention.

## Data Availability

The data are available on reasonable requests to the corresponding author but are restricted according to the decision by the ethical approval, Dnr number; 2017/43.

## Abbreviations

CBT: Cognitive Behavioural Therapy
MRC: Medical Research Council
NSPH: Swedish Partnership for Mental Health
PI: Principal Investigator
PTSD: Post Traumatic Stress Disorder
RCT: Randomized Controlled Trial
SAP: Sensory Awareness Program
SCP: Sensory Connection Program
TAU: Treatment As Usual
TIDieR: Template for Intervention Description and Replication
